# Mitigation of hysteresis due to a pseudo-photochromic effect in thermochromic smart window coatings

**DOI:** 10.1038/s41598-018-31519-x

**Published:** 2018-09-05

**Authors:** Christian Sol, Johannes Schläfer, Ivan P. Parkin, Ioannis Papakonstantinou

**Affiliations:** 1 University College London, Department of Electronic & Electrical Engineering, London, WC1E 7JE United Kingdom; 2 University College London, Department of Chemistry, London, WC1H 0AJ United Kingdom

## Abstract

The aim of thermochromic window coatings is to reduce the energy consumption in the built environment by passively switching between a high solar transmitting state at low temperatures and low solar transmitting state at high temperatures. Previous studies have highlighted the negative impact of phase transition hysteresis on the performance of reflection based thermochromic films. However in the literature, the best reported results have depended on vanadium dioxide nanoparticle composites and anti-reflective structures that modulate light via changes in absorption rather than reflection. In light of these factors, this work aims to demonstrate theoretically, how the effects of phase transition hysteresis and gradient differ between absorbing and non-absorbing thermochromic films. To quantify and compare the performance of films with different transition characteristics, we define a metric based on the varying net energy flux through the window over the course of a year, including thermal energy re-radiated into the building from the film. Specifically, and importantly for the field, we demonstrate that a pseudo-photochromic effect in absorbing thermochromic films mitigates the detrimental effects of phase transition hysteresis and gradient that have been reported for reflection based thermochromic films. We find that for moderate hysteresis widths of 15 °C, the performance of the non-absorbing case drops to ~60% of its initial value whilst the performance of the absorbing film only drops to ~95%. As a result we find that the absorbing case outperforms the non-absorbing case when hysteresis widths are greater than 8 °C.

## Introduction

**Figure 1:**
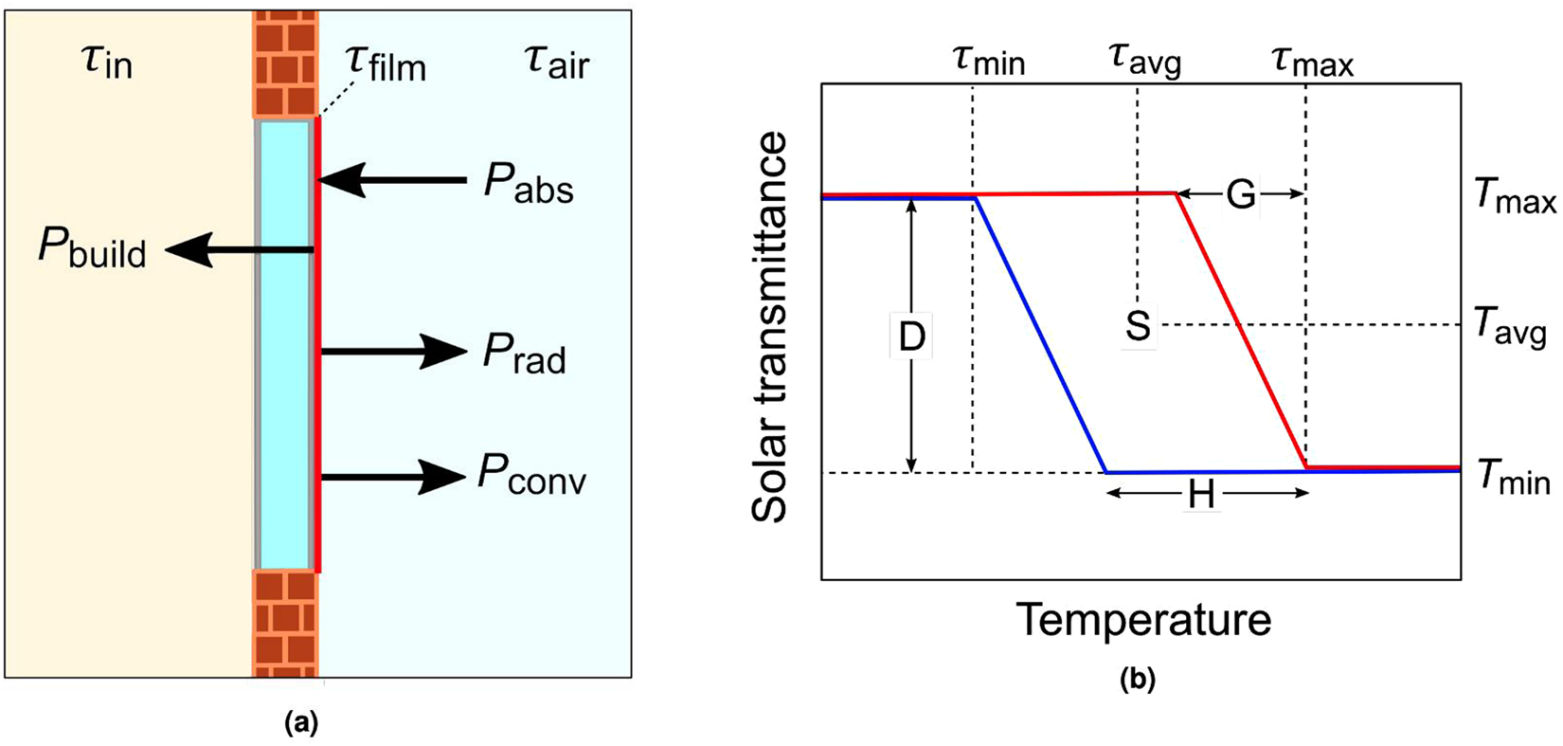
**(a)** Schematic of window energy fluxes with an absorbing coating on the outer surface of the window. **(b)** Schematic heating (red line) and cooling (blue line) hysteresis loop of a thermochromic film, including definitions of the transition gradient width G, hysteresis width H, switching temperature S, and modulation depth D.

Thermochromic window coatings have long held the promise of reducing heating and cooling energy loads within the built environment by passively modulating the transmission of solar radiation, switching between a high solar transmitting state at low temperatures and low solar transmitting state at high temperatures^1,2^. Previous works have aimed to model the energy saving performance of thermochromic coatings^3–5^ and others have presented idealised parameters for optimal performances^6^. Critical parameters that are often cited as key to improving performance are the switching temperature, transition gradient width, and hysteresis width^3^ (see Fig. 1b for definitions). Previous investigations into the effects of transition gradient width and hysteresis width have focused on idealised cases, where films modulate solar transmittance solely via changes in reflectance, such that they are non-absorbing and do not re-radiate thermal energy into the building^3,7^. In these cases it has been found that the switching temperature should be at room temperature, transition gradient sharp, and hysteresis width narrow. However, the performance of state-of-the-art thermochromic coatings depends largely on the use of anti-reflective structures^8–11^ or nanoparticles^12–16^ where modulation of solar transmittance is achieved primarily through changes in absorption rather than reflection, and specifically in the case of nanoparticles, where coatings exhibit significant hysteresis and gradient widths due to the small non-uniform domain sizes of the phase change components^13,14^. In light of these factors, this work aims to demonstrate for the first time, how the effects of transition gradient and hysteresis differ between absorbing and non-absorbing thermochromic coatings. The cause of these differences is found to be the additional dependence of the transition state on incident solar radiation, as imparted by solar absorption, which we refer to as a pseudo-photochromic effect. Importantly for the field, it is found that the negative effects of transition gradient and hysteresis widths are greatly mitigated as a result of solar absorption.

## Results and Discussion

The absorbing and non-absorbing coatings investigated are both assumed to have a solar transmittance modulation of 20% (*T*_max_ = 0.8; *T*_min_ = 0.6) which is a target theoretical performance for thermochromic window coatings^8,12^. In the non-absorbing case, as in previous work^3,7^, this modulation is assumed to occur by changes in reflectance, where the solar absorption is 0% in both high and low solar transmitting states. In the absorbing case, it is assumed that in the low temperature state 20% of incident solar radiation is absorbed whilst in the high temperature state 40% of incident solar radiation is absorbed, which is representative of optimised nanoparticle composites^12^.

Since the key benefit of a thermochromic window is that it can vary its properties in response to changing conditions it is important to compare annual performances. To do this we must determine the temperature and transmittance of the window coating as a function of time over the year. A schematic of the system investigated is shown in Fig. 1a. In all cases the thermochromic coating is assumed to be on the exterior surface of the window such that there is the greatest amount of thermal insulation between the coating and the inside of the building. The effect of this is to reduce thermal re-radiation from the film into the building, as is discussed later in the text. The variation in coating temperature *τ* over the year is described using a non-steady state heat transfer model, where the coating is assumed to be thin enough to be uniform in temperature. The change in temperature of the coating Δ*τ* is given by

1 $${\rm{\Delta }}\tau =\int [{P}_{{\rm{net}}}(t)/C]\,{\rm{d}}t=\int [({P}_{{\rm{abs}}}(t)-{P}_{{\rm{conv}}}(t)-{P}_{{\rm{build}}}(t)-{P}_{{\rm{rad}}}(t))/C]\,{\rm{d}}t,$$

where *P*_abs_, *P*_conv_, *P*_build_, and *P*_rad_, are the power absorbed from solar radiation, power convected to the outside air, power transfered into the building, and power radiated to the outside air respectively and where *C* is the heat capacity of the film. Mathematical definitions of these terms are given in the supplementary information. Since we are comparing absorbing and non-absorbing coatings it is necessary to consider the thermal energy transferred into the building from the absorbing coating. The reduction in performance due to thermal re-radiation is inversely proportional to the insulation quality of the window, since ${P}_{{\rm{build}}}\approx U({\rm{\Delta }}\tau )$ where Δ*τ* is the difference between film temperature and room temperature and *U* is the overall heat transfer coefficient of the window in W/m^2^.K. In this work we assume *U* = 2 W/m^2^.K, which is equivalent to a double glazed window with a low thermal emissivity inner coating^17^.

In the following, the transition characteristics i.e. switching temperature S, transition gradient width G, hysteresis width H, and modulation depth D, are defined as shown in Fig. 1b. A simplified model of the hysteretic dependence of film transmittance on temperature allows for a clear separation of the effects of hysteresis and transition gradient in our results. It is worth noting that performance of thermochromic films depends significantly on the location of implementation, however in the case of this work only one location (New York City) is discussed in the main text since the key results were found to be consistent between locations. The results from two further locations (London, Cairo) can be found in the supplementary information along with data describing the annual and diurnal variation in solar irradiance *I*_sol_, air temperature *τ*_air_ and other environmental parameters for each location.

To quantify and compare the performance of films with different transition characteristics we calculate the varying net energy flux Φ through the window over the course of a year, including thermal energy re-radiated into the building from the film. When the outside air temperature is below room temperature (constant 21 °C), we consider a high net energy flux to be beneficial, whereas when the outside air temperature is above room temperature a low net energy flux is considered beneficial. In both cases the beneficial net energy flux Φ_*B*_ is calculated in relation to a common reference taken to be non-absorbing with a static transmission set as the average of the high and low transmitting states *T*_avg_. The above is summarised in the equations below:

2 $${{\rm{\Phi }}}_{B}=(\begin{array}{cc}{I}_{{\rm{sol}}}(T-{T}_{{\rm{avg}}})+{P}_{\mathrm{build},s}-{P}_{\mathrm{build},r} & {\rm{for}}\,{\tau }_{{\rm{air}}} < 21{}^{^\circ }{\rm{C}}\\ {I}_{{\rm{sol}}}({T}_{{\rm{avg}}}-T)+{P}_{\mathrm{build},r}-{P}_{\mathrm{build},s} & {\rm{for}}\,{\tau }_{{\rm{air}}} > 21{}^{^\circ }{\rm{C}}\end{array}$$

where the subscripts *s* and *r* denote the study and reference values respectively. Integrated over the course of a year, equation (2) gives the total beneficial energy per unit area which we use as our metric to compare the performance of different films. Whilst this metric cannot be said to quantify the actual energy cost benefit of installing a thermochromic coating like in other studies^3,4^, it provides us with deep insights into the impact that the transition characteristics can have on the solar regulation of absorbing or non-absorbing thermochromic films. Additionally, focusing simply on calculating the annual variation in window energy flux allows for simulation times far quicker than for full building energy models, enabling a very large set of variations in parameters (>30,000 in this work) to be modeled.

**Figure 2:**
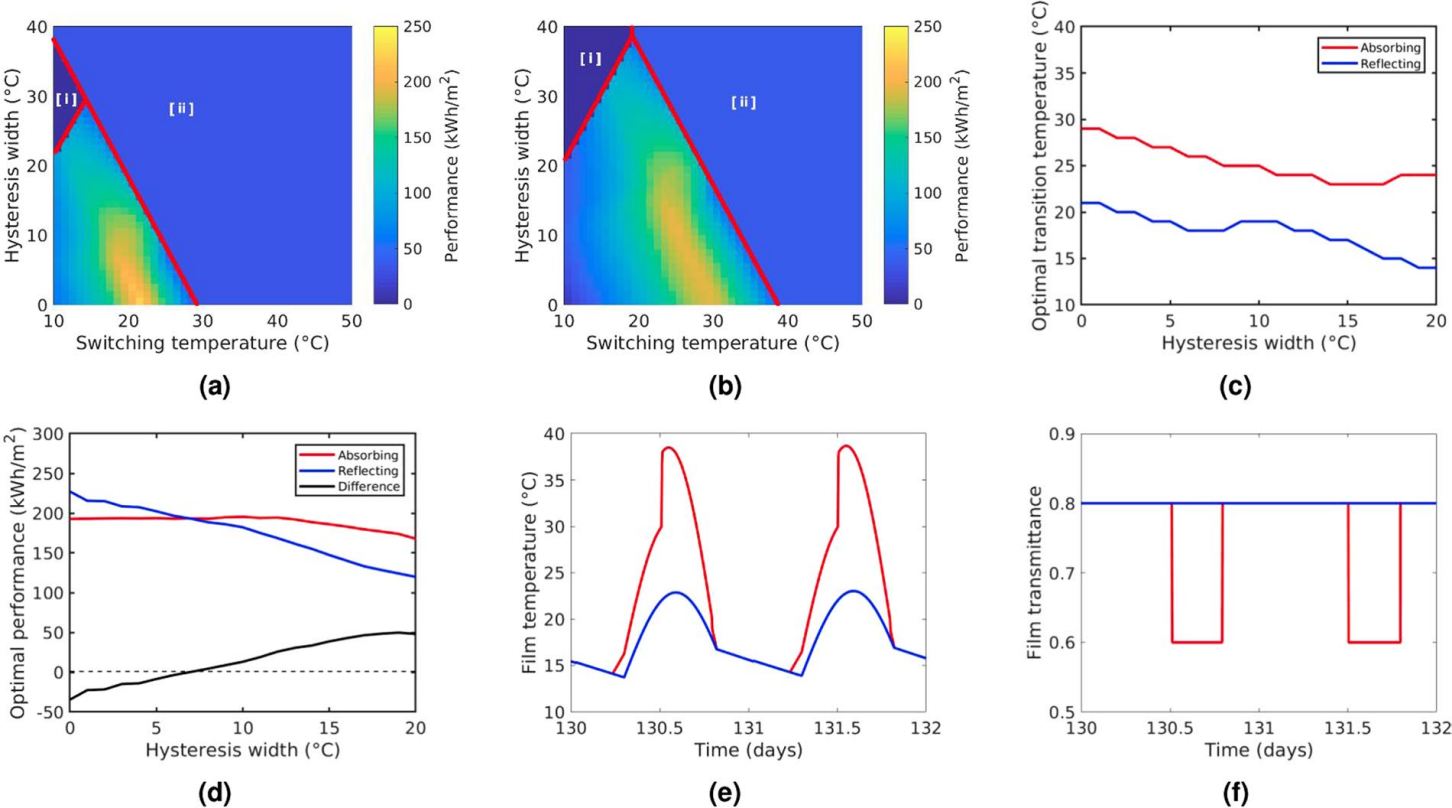
**(a,b)** Annually integrated performance metric from equation (2) of binary switching films (G = 0 °C) as a function of switching temperature and hysteresis width for **(a)** reflecting films and **(b)** absorbing films. We note that the large areas of uniform performance outlined in red towards the top left and right of figures (**a**,**b**) indicate the parameters for which the coatings are entirely in low [i] or high [ii] solar transmitting states. **(c)** Optimum transition temperature as a function of hysteresis width for absorbing and reflecting coatings. **(d)** Performance at the optimum transition temperature as a function of hysteresis width for absorbing and reflecting coatings. **(e,f)** Diurnal variation in **(e)** film temperature, and **(f)** film transmittance for absorbing and reflecting coatings at their respective optimum switching temperatures when H = 10 °C.

In Fig. 2 the annual performance as described in equation (2) is given as a function of switching temperature and hysteresis width for both reflecting (Fig. 2a) and absorbing (Fig. 2b) coatings with equal modulation depths and binary switching (G = 0 °C). As expected from previous literature^3^, in Fig. 2a we see that for the case of reflective films the optimum performance occurs when the switching temperature is at room temperature and that the performance rapidly diminishes with increasing hysteresis width. In contrast, for the case of absorbing films an immediate observation from Fig. 2c is that the optimum switching temperature is higher than for reflecting films, which can be expected since the equilibrium temperature under solar illumination will be significantly higher with increased solar absorption (see Fig. 2e). With regards to the increased optimum switching temperature it is important to note that the switching temperature of pure vanadium dioxide is around 68 °C, making it impractical for building temperature regulating applications. The switching temperature can be reduced with elemental doping (e.g W, Mo, Nb) however this comes at a cost of solar modulation depth^2^. This result suggests that absorbing films would require less elemental doping than those which are purely reflective and thus preserve more of the solar modulation depth achievable with undoped vanadium dioxide. As described in previous works^6^, we note that the optimum performance for the absorbing case for hysteresis widths of 0 °C is lower than the reflecting case since some of the absorbed energy is transferred into the building as thermal energy rather than being reflected outwards. However, in Fig. 2d, we see that the rapid decline in performance with increasing hysteresis is mitigated for absorbing films, with high performances still seen for hysteresis widths greater than 15 °C. In Fig. 2d we compare the performances absorbing and reflecting films when both are at their respective optimum switching temperatures as a function of hysteresis width. It can be seen from Fig. 2d that for moderate hysteresis widths of 15 °C, the performance of the non-absorbing case drops to ~60% of its initial value whilst the performance of the absorbing film only drops to ~95%. As a result we find that the absorbing case outperforms the non-absorbing case when hysteresis widths are greater than 8 °C. The reason for this can be appreciated from Fig. 2e where we see that the absorbing film reaches a significantly wider range of temperatures due to the additional dependence of film temperature on solar irradiance. The increased range of temperatures allows for the absorbing film to remain responsive throughout more of the year despite moderately large hysteresis widths. This can be seen in Fig. 2e where film transmittance is plotted over a period of two days. We see that due to the wider range of temperatures reached, the absorbing film remains responsive despite the hysteresis width of 10 °C. In contrast, during the same period, the non-absorbing film is prevented from switching and loses its functionality as a result of hysteresis.

**Figure 3:**
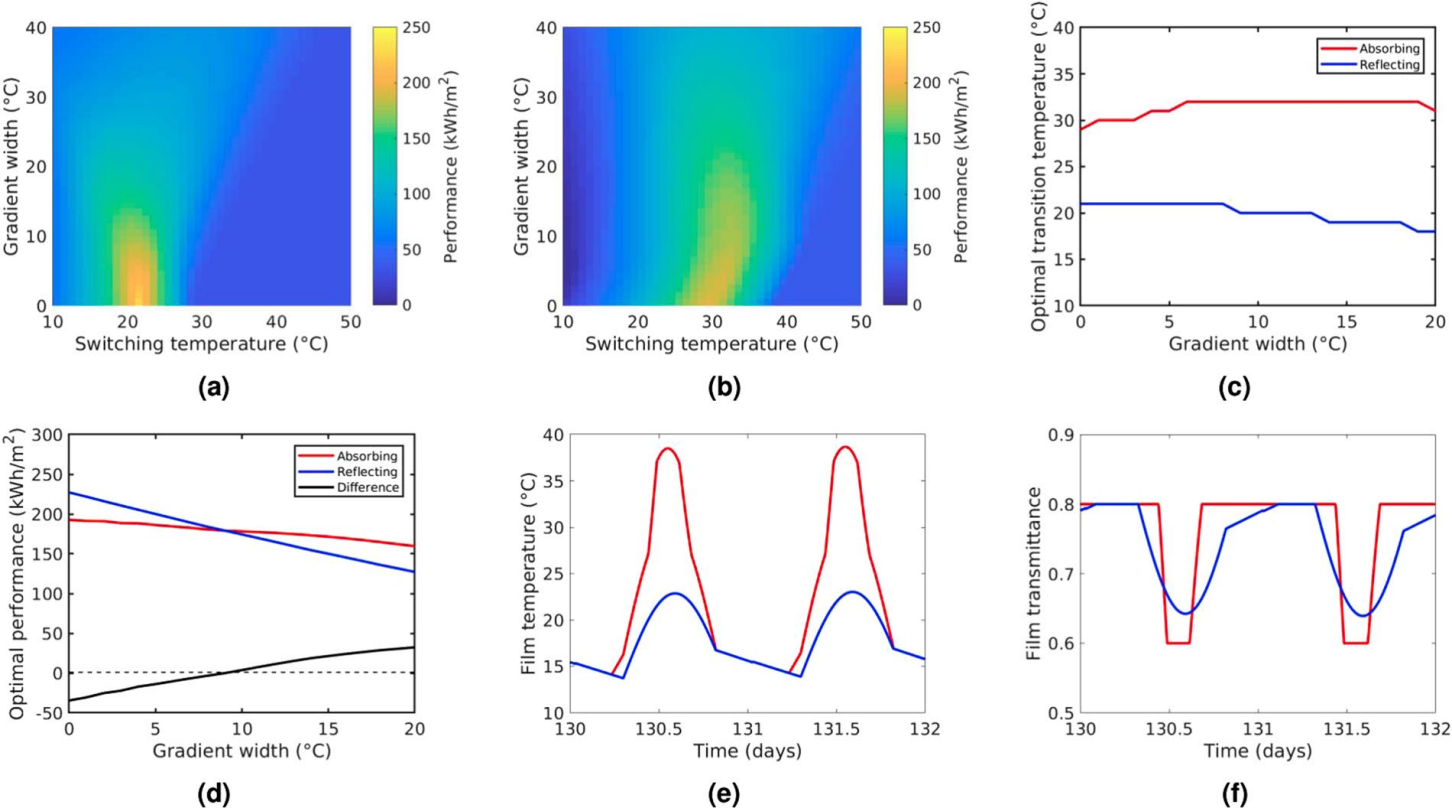
**(a,b)** Annually integrated performance metric from equation (2) of graded films (H = 0 °C) as a function of switching temperature and gradient width for **(a)** reflecting films and **(b)** absorbing films. **(c)** Optimum transition temperature as a function of gradient width for absorbing and reflecting coatings. **(d)** Performance at the optimum transition temperatures as a function of gradient width for absorbing and reflecting coatings. **(e,f)** Diurnal variation in **(e)** film temperature, and **(f)** film transmittance for absorbing and reflecting coatings at their respective optimum switching temperatures when G = 10 °C.

The difference in performance between the two types of modulation is made more significant by the introduction of a gradient in the transition. In Fig. 3 the performance metric is given as a function of switching temperature and gradient width for both absorbing and reflecting coatings (H = 0 °C). For the reflecting film in Fig. 3a, as reported previously^3^, the increase in transition gradient results in a rapid loss of performance. Specifically in our case, as shown in Fig. 3d, there is loss in performance of ~50% for gradient widths greater than 20 °C. In the case of the absorbing film in Fig. 3b the loss of performance for gradient widths of 20 °C is less than 25% of its original performance. As a result of this, we see that when gradient widths are greater than 10 °C, the performances of absorbing films are higher than that of reflecting films when both are at their respective optimum switching temperatures. The reasoning for this can be seen in Fig. 3f where in the non-absorbing case, the increase in gradient results in films only partially switching between high and low temperature states and a loss in performance. The introduction of a gradient in the transition is less detrimental for absorbing films due to the interdependence of coating temperature and absorption, which results in a feedback loop that drives the transition further towards the extremes, resulting in a much sharper transition than in the reflecting case.

## Conclusions

In summary, the detrimental effects of phase transition hysteresis on performance that are apparent in non-absorbing thermochromic coatings are greatly mitigated in absorbing coatings due to an additional dependence of transition state on the intensity of incident solar radiation. This pseudo-photochromic effect is further enhanced in coatings with sloping transitions between high and low temperature states, as commonly seen in nanoparticulate vanadium dioxide films. The results presented in this work can be seen as encouraging to all those pursuing vanadium dioxide window coatings for passive solar control applications. Specifically, the detrimental effects of increased hysteresis and transition gradient widths have been shown to be significantly mitigated in the case of absorbing thermochromic films, provided that the coated windows have sufficiently high insulation values to prevent absorbed solar radiation being transmitted into the building. Additionally, absorbing thermochromic coatings are found to have higher optimal transition temperatures compared with purely reflective coatings, such that significantly less doping may be required to give an optimal switching temperature. Although the majority of the discussion in this work has focused specifically on vanadium dioxide, these key results can be generalised to any smart window coatings that make use of absorbing thermochromic materials. Whilst this work has demonstrated the significant differences between the switching dynamics of absorbing and non-absorbing films with varying degrees of transition hysteresis and gradient, it would be interesting in the future to investigate the effect of these differences on the overall reduction of heating and cooling costs within the built environment by employing a full building energy simulation, as this has so far been overlooked in the literature.

## Methods

Calculations of coating temperature as a function of time were performed using a 1D non-steady state heat transfer model written in MATLAB with incremental changes in temperature calculated as described in equation (1). A time resolution of 10 s was chosen for the model by convergence testing. The thermochromic hysteresis effect was modeled using a set of logic conditions that depend on the coating temperature. As described in the text, in all cases the absorbing and non-absorbing coatings investigated have a solar transmittance modulation of 20% (*T*_max_ = 0.8; *T*_min_ = 0.6). In the non-absorbing case this modulation is assumed to occur by changes in reflectance, where the solar absorption is 0% in both high and low solar transmitting states. In the absorbing case, it is assumed that in the low temperature state 20% of incident solar radiation is absorbed whilst in the high temperature state 40% of incident solar radiation is absorbed.

## Electronic supplementary material

Supplementary Information

## Data Availability

All data and associated protocols are available upon reasonable request.
