# Supplementary material for: Mitigation of hysteresis due to a pseudo-photochromic effect in thermochromic smart window coatings
Source: Sci Rep. 2018 Sep 5;8:13249. doi: 10.1038/s41598-018-31519-x (PMC6125580; doi:10.1038/s41598-018-31519-x)
Supplement: Supplementary file 1 — Supplementary Information [file 41598_2018_31519_MOESM1_ESM.pdf]

Mitigation of hysteresis due to a pseudo-photochromic effect in thermochromic smart window coatings: Supplementary information

Christian Sol, Johannes Schläfer, Ivan P. Parkin, Ioannis Papakonstantinou

1 Window energy flux definitions

The energy flux P_{net} into the window film as described in the main text can be given by,

$$P_{\mathrm{net}} = P_{\mathrm{abs}} - P_{\mathrm{rad}} - P_{\mathrm{conv}} - P_{\mathrm{build}} \tag{1}$$

where P_{sol} is the solar power absorbed by the film, P_{rad} is the radiation emitted from the film to the outside air, P_{conv} is the power transferred to the ambient air by convection, and P_{build} is the power transferred into the building.

The components P_{sol}, P_{rad}, P_{conv}, and P_{build} are given by:

$$P_{\mathrm{abs}} = I_{\mathrm{sol}} A_{\mathrm{sol}}(\tau) \tag{2}$$

$$P_{\mathrm{rad}} = I_{\mathrm{BB}}(\tau)\epsilon_{\mathrm{out}} \tag{3}$$

$$P_{\mathrm{conv}} = h(\tau - \tau_{\mathrm{out}}) \tag{4}$$

$$P_{\mathrm{build}} = U(\tau - \tau_{\mathrm{in}}) + I_{\mathrm{BB}}(\tau)\,(1 - \epsilon_{\mathrm{out}}) \tag{5}$$

where I_{sol} is the solar irradiance at the window surface A_{sol} is absorption of the film integrated over the AM1.5 solar spectrum, where $I_{\mathrm{BB}}(\tau)$ is the spectral emission from a black body at temperature τ and ϵ_{out} defines portion of the thermal energy that is emitted outwards from the building by the film, where τ is the temperature of the film and τ_{in} and τ_{out} are the internal and outside temperatures respectively, and where U and h are heat transfer coefficients in Watts/°C. The thermal emissivity of the window can be approximated as constant for nanoparticulate vanadium dioxide coatings, since changes in absorption are confined to visible and near infrared wavelengths[4, 6]. The convective heat transfer coefficient h is given commonly for building applications as a function of wind speed by[3],

$$\begin{aligned} h &= 4.0v_\infty + 5.6 \qquad v_\infty < 5\mathrm{ms}^{-1} \\ h &= 7.1v_\infty^{0.78} \qquad v_\infty \geq 5\mathrm{ms}^{-1} \end{aligned} \tag{6}$$

where v_∞ is the free stream wind speed; wind speed values were interpolated over the year from monthly averages taken from ref[1].

2 Environmental parameters

The annual and diurnal variations in solar irradiance for the different locations reported in this work were derived following ref [5]. The terrestrial solar irradiance arriving at the surface of the earth is given relative to the extraterrestrial solar irradiance by

$$I_t = \alpha I_{et}$$

where α an empirically derived coefficient representing solar attenuation in different climates [2]. In the case of this work, the value of α for each location was determined by fitting calculated peak values to measured peak solar irradiance found from ref [1].

The hourly variation in extraterrestrial solar irradiance over a year is given by,

$$I_{et} = 1370\epsilon_0 \sin(\beta)$$

where the empirical coefficient of annual variation ϵ_0 is given by,

$$\epsilon_0 = 1 + 0.033\,[2\pi(t_d - 10)/365]$$

and the hourly variation, i.e. the elevation of the sun relative to the surface of the earth, is given by

$$\beta = \arcsin(a + b\cos(\tau))$$

where τ is the solar hour, given as a function of the hour of the day t_h by

$$\tau = \frac{\pi}{12}(t_h - 12)$$

and where a and b are given by,

$$a = \sin(\lambda)\sin(\delta)$$
$$b = \cos(\lambda)\cos(\delta)$$

where λ is the latitude of the observer and δ is the solar declination given by,

$$\delta = -0.4093\cos\,[2\pi(t_d + 10)/365]$$

where t_d is the day of the year.

The diurnal variation of temperature can be split into three sections and is defined in relation to the time of sunrise and sunset along with values for the minimum and maximum temperatures reached taken from ref[1]. The sunset and sunrise times are given by

$$t_{ss} = 12 + \frac{12}{\pi}\arccos\left(-\frac{a}{b}\right)$$
$$t_{sr} = 12 - \frac{12}{\pi}\arccos\left(-\frac{a}{b}\right)$$

whilst the outside air temperature is then given by,

$$\tau_{\text{set}} + \frac{(\tau_{\min} - \tau_{\text{set}})(24 + t_h - t_{ss})}{(t_{sr} + 1.5) + (24 - t_{ss})} \qquad \text{for stage I}$$
$$\tau_{\min} + (\tau_{\max} - \tau_{\min}\sin\left[\frac{\pi(t_h - t_{sr} - 1.5)}{t_{ss} - t_{sr}}\right] \qquad \text{for stage II}$$
$$\tau_{\text{set}} + \frac{(\tau_{\min} - \tau_{\text{set}})(t_h - t_{ss})}{(t_{sr} + 1.5) + (24 - t_{ss})} \qquad \text{for stage III}$$

where τ_{set} is the outside temperature at sunset for the previous day. The initial value for this parameter is predetermined by a preliminary simulation of the solar irradiance and outside temperature over the year. At the start of a simulation it is set at the predetermined initial value, after which it is be updated daily at $t_h = t_{ss}$.

3 Extra locations

As reported previously, in the case of more extreme climates where temperatures are almost always above (see Cairo) or almost always below (see London) room temperature during daylight hours thermochromic window coatings are much less beneficial since the peak performance is actually very similar to the static none switching cases. In the low temperatures of London it would be almost equally beneficial to have a static high solar transmitting window whereas in the high temperatures of Cairo it would be similarly beneficial to have a static low solar transmitting window. In spite of this, as described in the main text, we do still see that the decreased susceptibility to the effects of hysteresis and gradient, and increased optimal transition temperatures are maintained in each location when comparing absorbing and non-absorbing films.

3.1 London

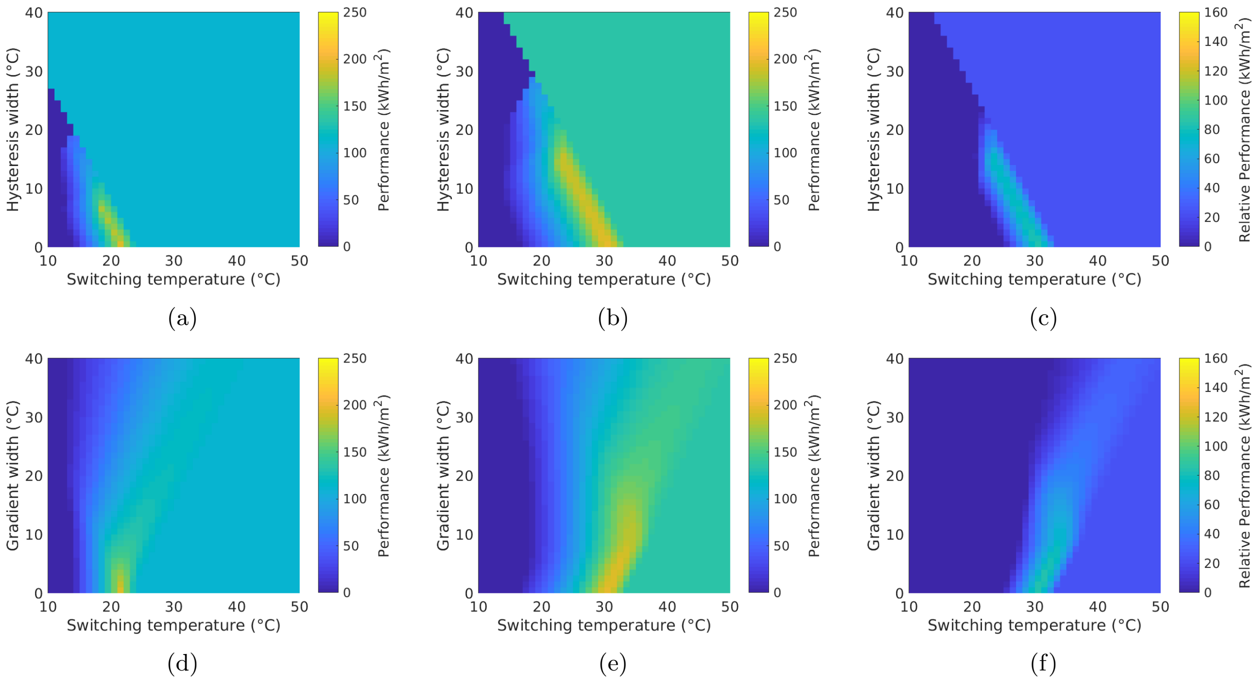

Figure 1: London study; **(a-c)** Annual performance of binary films (G = 0°C) as a function of switching temperature and hysteresis width for **(a)** reflecting films **(b)** absorbing films and **(c)** difference of absorbing subtracted by reflecting films; $T_{\mathrm{max}} = 0.8$; $T_{\mathrm{min}} = 0.6$. **(d-f)** Annual performance of graded films (H = 0°C) as a function of switching temperature and gradient width for **(d)** reflecting films **(e)** absorbing films and **(f)** difference of absorbing subtracted by reflecting films; $T_{\mathrm{max}} = 0.8$; $T_{\mathrm{min}} = 0.6$.

3.2 Cairo

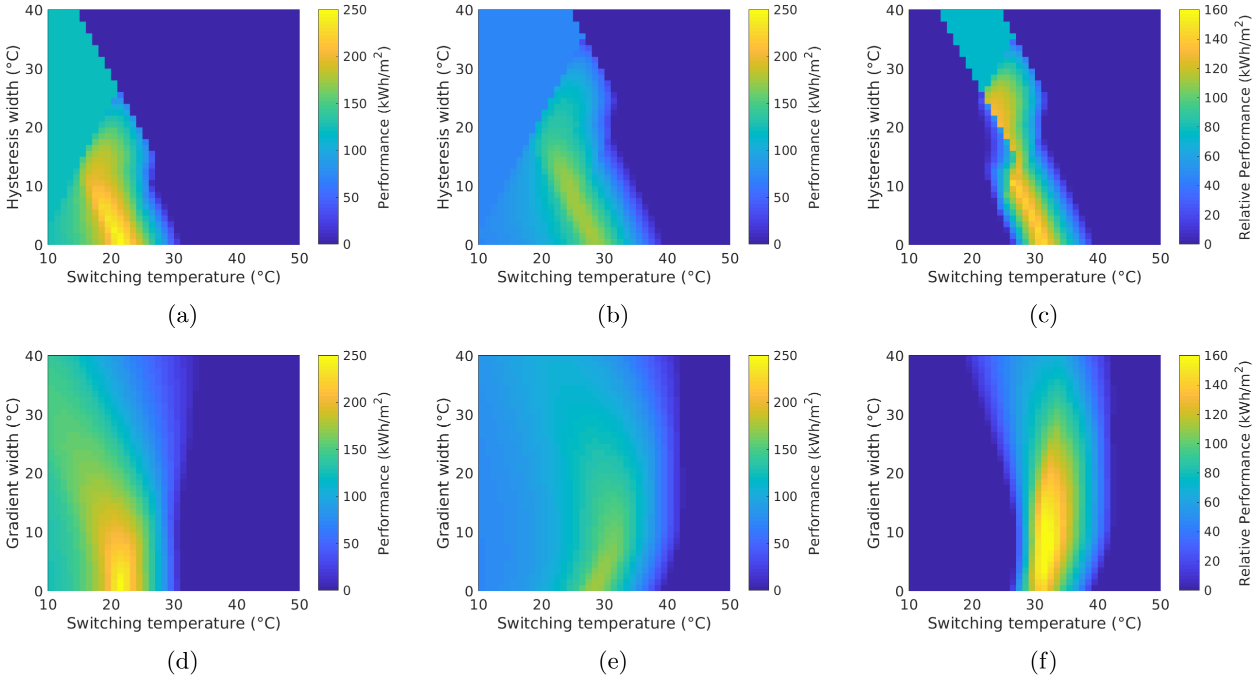

Figure 2: Cairo study; **(a-c)** Annual performance of binary films (G = 0°C) as a function of switching temperature and hysteresis width for **(a)** reflecting films **(b)** absorbing films and **(c)** difference of absorbing subtracted by reflecting films; $T_{\mathrm{max}} = 0.8$; $T_{\mathrm{min}} = 0.6$. **(d-f)** Annual performance of graded films (H = 0°C) as a function of switching temperature and gradient width for **(d)** reflecting films **(e)** absorbing films and **(f)** difference of absorbing subtracted by reflecting films; $T_{\mathrm{max}} = 0.8$; $T_{\mathrm{min}} = 0.6$.

References

[1] Drury B Crawley, Curtis O Pedersen, Linda K Lawrie, and Frederick C Winkelmann. Energyplus: energy simulation program. *ASHRAE journal*, 42(4):49, 2000.

[2] J Glover and JSG McCulloch. The empirical relation between solar radiation and hours of sunshine. *Quarterly Journal of the Royal Meteorological Society*, 84(360):172–175, 1958.

[3] Suresh Kumar and SC Mullick. Wind heat transfer coefficient in solar collectors in outdoor conditions. *Solar Energy*, 84(6):956–963, 2010.

[4] S-Y Li, Gunnar A Niklasson, and Claes-Göran Granqvist. Nanothermochromics: calculations for VO_2 nanoparticles in dielectric hosts show much improved luminous transmittance and solar energy transmittance modulation. *Journal of Applied Physics*, 108(6):063525, 2010.

[5] John Monteith and Mike Unsworth. *Principles of environmental physics*. Academic Press, 2007.

[6] Yijie Zhou, Aibin Huang, Yamei Li, Shidong Ji, Yanfeng Gao, and Ping Jin. Surface plasmon resonance induced excellent solar control for VO_2@SiO_2 nanorods-based thermochromic foils. *Nanoscale*, 5(19):9208–9213, 2013.
